# Overexpression of Interleukin-8 Promotes the Progression of Fatty Liver to Nonalcoholic Steatohepatitis in Mice

**DOI:** 10.3390/ijms242015489

**Published:** 2023-10-23

**Authors:** Ye Eun Cho, Yeonsoo Kim, Seung-Jin Kim, Haeseung Lee, Seonghwan Hwang

**Affiliations:** 1 College of Pharmacy and Research Institute for Drug Development, Pusan National University, Busan 46241, Republic of Korea; eloss98@pusan.ac.kr (Y.E.C.); sum270@pusan.ac.kr (Y.K.); haeseung@pusan.ac.kr (H.L.); 2 Department of Biochemistry, College of Natural Sciences, Kangwon National University, Chuncheon 24341, Republic of Korea; sjk@kangwon.ac.kr; 3 Laboratory of Liver Diseases, National Institute on Alcohol Abuse and Alcoholism, National Institutes of Health, Bethesda, MD 20892, USA

**Keywords:** fatty liver, nonalcoholic steatohepatitis, chemokine, neutrophil, interleukin-8

## Abstract

Nonalcoholic steatohepatitis (NASH) is an advanced stage of fatty liver disease characterized by liver damage, inflammation, and fibrosis. Although neutrophil infiltration is consistently observed in the livers of patients with NASH, the precise role of neutrophil-recruiting chemokines and infiltrating neutrophils in NASH pathogenesis remains poorly understood. Here, we aimed to elucidate the role of neutrophil infiltration in the transition from fatty liver to NASH by examining hepatic overexpression of interleukin-8 (IL8), a major chemokine responsible for neutrophil recruitment in humans. Mice fed a high-fat diet (HFD) for 3 months developed fatty liver without concurrent liver damage, inflammation, and fibrosis. Subsequent infection with an adenovirus overexpressing human IL8 for an additional 2 weeks increased IL8 levels, neutrophil infiltration, and liver injury in mice. Mechanistically, IL8-induced liver injury was associated with the upregulation of components of the NADPH oxidase 2 complex, which participate in neutrophil oxidative burst. IL8-driven neutrophil infiltration promoted macrophage aggregate formation and upregulated the expression of chemokines and inflammatory cytokines. Notably, IL8 overexpression amplified factors associated with fibrosis, including collagen deposition and hepatic stellate cell activation, in HFD-fed mice. Collectively, hepatic overexpression of human IL8 promotes neutrophil infiltration and fatty liver progression to NASH in HFD-fed mice.

## 1. Introduction

Nonalcoholic fatty liver disease (NAFLD) encompasses a range of conditions, from fatty liver to nonalcoholic steatohepatitis (NASH), cirrhosis, and hepatocellular carcinoma [1,2]. NAFLD is considered the primary cause of chronic liver disease owing to the increasing prevalence of metabolic syndromes [3,4]. Fatty liver, which is characterized by excessive fat accumulation in the liver, has been estimated to affect approximately 30% of the adult population [5,6]. Although fatty liver rarely impairs liver function, approximately 20% of individuals with fatty liver progress to NASH, a more severe form of the disease characterized by hepatocellular injury, inflammation, and fibrosis [7]. As NASH irreversibly progresses into cirrhosis and carcinoma, the transition from fatty liver to NASH, an early stage of NAFLD development, is considered a target for NAFLD treatment [8]. Accordingly, it is crucial to understand the drivers of inflammation in NAFLD, as inflammation plays a crucial role in promoting NASH through various factors, including cytokines, chemokines, and immune cells such as monocytes and neutrophils.

Neutrophils are polymorphonuclear leukocytes that serve as the first line of defense against infection through mechanisms such as phagocytosis, reactive oxygen species (ROS) production, degranulation, proteolysis, and neutrophil extracellular trap formation [9,10,11,12,13]. Neutrophils have been implicated in promoting tissue damage and inflammation during NASH development [14], and emerging evidence suggests a close association between neutrophils and NASH progression. The neutrophil-to-lymphocyte ratio (NLR) has been shown to be correlated with the severity of hepatocyte ballooning, lobular inflammation, and fibrosis in patients with NAFLD [15]. Additionally, Yilmaz et al. have reported that the NLR is proportional to the NAFLD activity score and could function as a marker of NASH severity [16]. Furthermore, hepatic neutrophil infiltration is a distinct characteristic of NASH and is not observed in fatty liver [17,18,19].

In humans, neutrophil chemotaxis is induced by chemokines, such as C-X-C motif chemokine ligand (CXCL)1, CXCL2, CXCL6, and interleukin (IL)-8 (also known as CXCL8) [20]. Neutrophils express chemokine receptors, such as CXCR1 and CXCR2. CXCR1 functions as a receptor for CXCL6 and IL8, while CXCR2 interacts with CXCL1, CXCL2, CXCL6, and IL8 [21,22]. Compared with the livers of patients with fatty liver, the livers of patients with NASH exhibit upregulated expression of neutrophil-recruiting chemokines, such as CXCL1 and their receptors (CXCR1 and CXCR2) [17]. Moreover, increased expression of the adhesion molecule SELE, which facilitates neutrophil infiltration into vasculature, has been observed in the livers of patients with NASH [17]. These findings suggest the involvement of neutrophil infiltration in the progression of fatty liver to NASH in humans. However, the role of neutrophils in fatty liver-to-NASH progression remains unclear, owing to a lack of suitable animal models. Notably, neutrophil biology differs between humans and mice. For example, the number of blood neutrophils in mice is approximately 25% of that in humans (1 × 10^9^/L vs. 4 × 10^9^/L) [23,24]. Moreover, the mouse genome does not possess a homolog of the human *IL8* gene, and mouse hepatocytes fail to efficiently induce Cxcl1 expression under inflammatory conditions, unlike human hepatocytes [25]. Accordingly, these differences may contribute to the resistance of obese mice to NASH development, as neutrophil recruitment is less effective in these mice.

Recently, Cxcl1 overexpression in the livers of obese mice was shown to promote neutrophil infiltration and progression of fatty liver to NASH [26]. However, the role of IL8, the most potent neutrophil-attracting chemokine in humans [14], has not been directly examined in the context of NASH development, owing to the absence of an *IL8* homolog in mice. Although our previous paper has suggested that IL8 might enhance the NASH-inducing effect of Cxcl1 overexpression in mice, it has not been tested whether IL8 may independently promote neutrophil infiltration or NASH progression in mice [25]. To address this, we overexpressed the human *IL8* gene in high-fat diet (HFD)-fed mice using an adenovirus engineered to express the human *IL8* gene, which induced increased neutrophil infiltration into the liver. IL8-overexpressing mice exhibited elevated tissue injury, oxidative stress, inflammation, and fibrosis, resembling the characteristics of NASH. Herein, we provide experimental data suggesting that IL8 overexpression and subsequent neutrophil infiltration in the liver could facilitate the progression of fatty liver to NASH in mice. Furthermore, combining HFD feeding with IL8 overexpression can serve as a useful tool to explore neutrophil-driven NASH in mice.

## 2. Results

### 2.1. Hepatic IL8 Transcript Levels Are Proportional to NASH Severity

**Figure 1:**
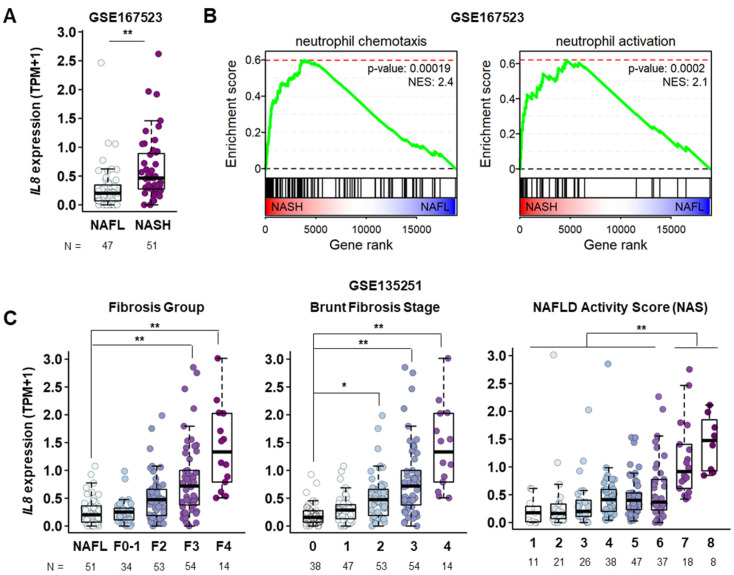
Elevated IL8 transcript levels in the course of NAFLD progression. (**A**) mRNA expression levels of the *IL8* gene in the livers of patients with fatty liver (*n* = 47) and NASH (*n* = 51) from a publicly available RNA sequencing database (GSE167523). Statistical significance was assessed using Student’s *t*-tests (** *p* < 0.01). (**B**) GSEA plots showing the enrichment of genes associated with neutrophil chemotaxis and neutrophil activation in gene rank based on differential expression between NASH vs. NAFL livers. (**C**) mRNA expression levels of the *IL8* gene in the liver among different stages of NAFLD obtained from a publicly available RNA sequencing database (GSE135251). F0–F1, F2, F3, and F4 represent different NASH groups classified by the severity of fibrosis. TPM refers to transcripts per million base pairs. For comparisons involving more than three groups, a one-way analysis of variance (ANOVA) was conducted, followed by post-hoc Tukey’s tests to determine specific group differences. (* *p* < 0.05, ** *p* < 0.01). IL8, interleukin 8; GSEA, gene set enrichment analysis; NAS, NAFLD activity score; NAFLD, nonalcoholic fatty liver disease; NASH, nonalcoholic steatohepatitis.

To determine the dynamic changes in IL8 expression during NASH progression, we analyzed publicly available RNA sequencing (RNA-seq) data from patients with NAFLD (GSE135251 and GSE167523), which have been recently published with the information for hepatic gene expression compared between individuals with different stages of NASH as well as fatty liver [27,28]. The liver tissues of patients with NASH showed elevated *IL8* transcript levels compared to those of patients with fatty liver (Figure 1A). Subsequent functional enrichment analyses revealed significant upregulation of genes involved in the regulation of leukocyte migration, encompassing neutrophil chemotaxis and activation, in the livers of patients with NASH (Figure 1B, Supplementary Figure S1). This observation is consistent with the role of IL8 in mediating neutrophil recruitment and behavior during an inflammatory response [18]. Furthermore, *IL8* transcript levels progressively increased with NASH advancement, as indicated by the fibrosis stage, NAFLD activity score, and Brunt fibrosis grade (Figure 1C). Taken together, these findings indicate a possible association between the increase in IL8 expression, infiltration, activation of neutrophils, and NASH progression.

### 2.2. Adenovirus-Mediated Overexpression of IL8 Promotes Neutrophil Infiltration and Tissue Injury in the Livers of HFD-Fed Mice

**Figure 2:**
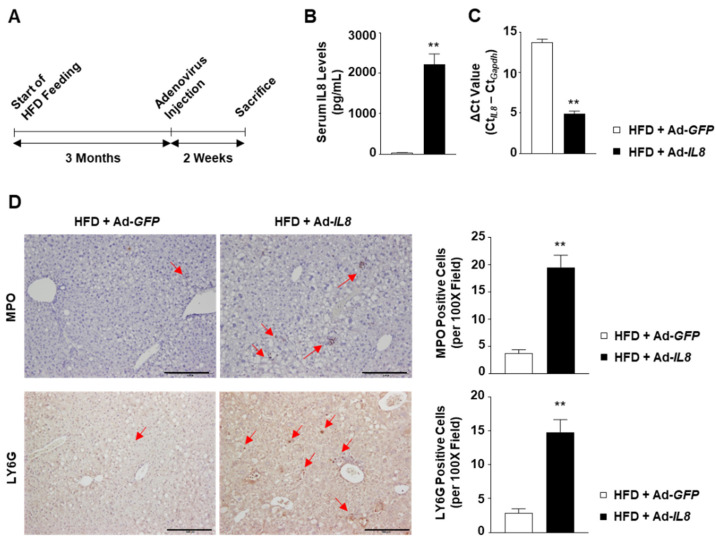
*IL8* overexpression induces increased hepatic neutrophil infiltration in the livers of HFD-fed mice. C57BL/6 mice fed an HFD for 3 months were infected with an adenovirus overexpressing the human *IL8* gene (Ad-*IL8*) or an adenovirus expressing green fluorescence protein (Ad-*GFP*) via the tail vein and sacrificed for analysis 2 weeks post-infection. (**A**) Schematic illustration of the experimental design. (**B**) Serum IL8 levels measured using ELISA analysis. (**C**) Hepatic *IL8* transcript levels were calculated using the ΔCt method (Ct_IL8_ – Ct_Gapdh_). (**D**) Paraffin-embedded liver sections were subjected to immunohistochemical analysis of MPO and LY6G (*n* = 5/group). Red arrows indicate MPO- or LY6G-positive cells. Scale bars indicate 200 μm. The number of MPO- or LY6G-positive cells per 100× field was counted and illustrated in the graph on the right-hand side. Values represent the mean ± standard error of the mean (SEM). Statistical evaluation was performed using Student’s *t*-tests (** *p* < 0.01). ELISA, enzyme-linked immunosorbent assay; HFD, high-fat diet; IL8, interleukin 8; MPO, myeloperoxidase.

As elevated IL8 expression is correlated with NASH severity, we subsequently examined the potential role of IL8 in NASH progression. Accordingly, C57BL/6 mice fed an HFD for 3 months to induce fatty liver were subsequently infected with an adenovirus overexpressing the human *IL8* gene (Ad-*IL8*) or a control virus expressing *GFP* (Ad-*GFP*) (Figure 2A). Tail vein injection of Ad-*IL8* increased serum IL8 levels up to 2200 pg/mL at 2 weeks post-infection (Figure 2B). Furthermore, reverse transcription-quantitative polymerase chain reaction (RT-qPCR) analysis revealed that Ad-*IL8* infection resulted in the overexpression of human IL8 in the livers of mice (Figure 2C). Immunohistochemical analysis revealed that hepatic IL8 overexpression increased the number of cells positive for either of two neutrophil-specific markers, myeloperoxidase (MPO) or LY6G, in the liver (Figure 2D). Collectively, these findings indicate that IL8 overexpression could induce hepatic neutrophil infiltration.

### 2.3. IL8 Overexpression Increases Oxidative Stress in the Livers of HFD-Fed Mice

**Figure 3:**
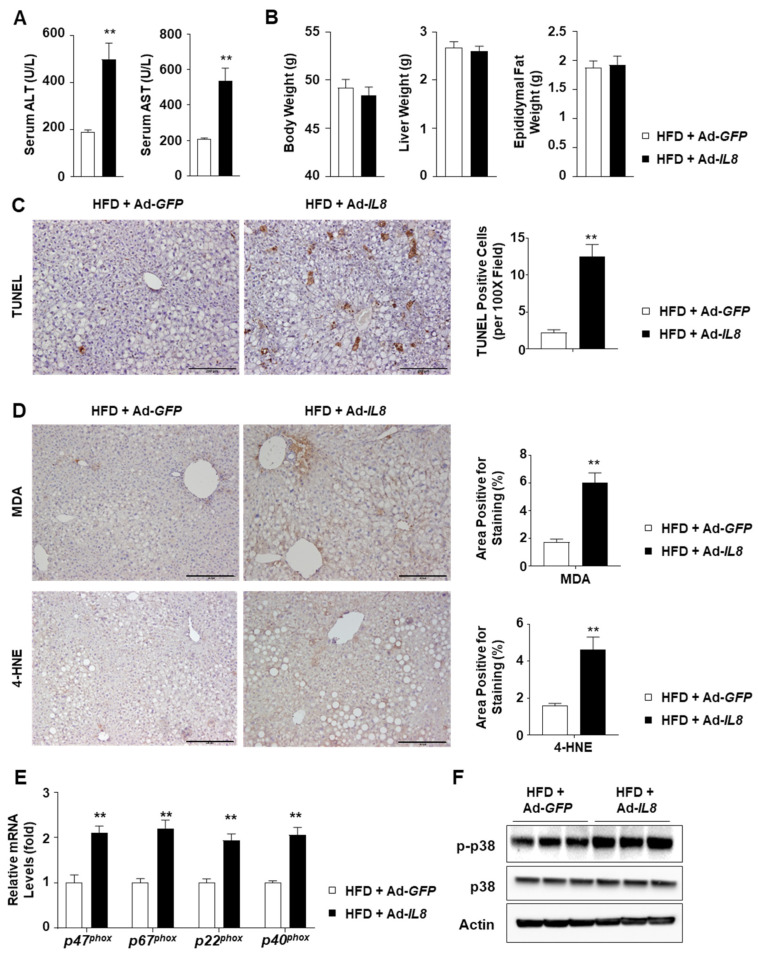
IL8 overexpression enhances liver injury and oxidative stress in the livers of HFD-fed mice. (**A**) Serum ALT and AST levels (*n* = 5/group). (**B**) Body weight and liver weight (*n* = 5/group). (**C**,**D**) Paraffin-embedded liver sections were subjected to the TUNEL assay (panel **C**) and immunohistochemical analyses of MDA and 4-HNE (panel **D**) (*n* = 5/group). Representative images of each staining are presented (**left**). Scale bars indicate 200 μm. Quantification of the area positive for each staining is illustrated in graphs (**right**). (**E**) Mouse liver homogenates were subjected to RT-qPCR analysis of the genes involved in NOX2 complex formation and neutrophil oxidative burst. (*n* = 5/group). (**F**) Immunoblot analysis of mouse liver homogenates. Values represent the mean ± standard error of the mean (SEM). Statistical evaluation was performed using Student’s *t*-tests (** *p* < 0.01). 4-HNE, 4-hydroxynonenal; ALT, alanine transaminase; AST, aspartate transaminase; HFD, high-fat diet; IL8, interleukin 8; MDA, malondialdehyde; NOX2, NADPH oxidase 2; RT-qPCR, reverse transcription-quantitative polymerase chain reaction.

Subsequently, we analyzed the liver injury parameters to determine the consequences of hepatic IL8 overexpression and subsequent neutrophil recruitment. IL8 overexpression exacerbated liver injury, as indicated by elevated serum alanine transaminase (ALT) and aspartate transaminase (AST) levels (Figure 3A), whereas the body, liver, and epididymal fat weights were unaffected (Figure 3B). IL8 overexpression-induced exacerbated liver injury was further confirmed by terminal deoxynucleotidyl transferase dUTP nick-end labeling (TUNEL) (Figure 3C) as well as hematoxylin and eosin staining (Supplementary Figure S2). To elucidate the potential mechanism underlying IL8-induced liver injury, we conducted immunohistochemical analyses of malondialdehyde (MDA) and 4-hydroxynonenal (4-HNE), both of which are known indicators of lipid peroxidation that form adducts with proteins [29,30]. IL8 overexpression increased the MDA- and 4-HNE-positive areas in the livers of HFD-fed mice (Figure 3D). Neutrophil oxidative burst is a critical process that contributes to ROS production by neutrophils. NADPH oxidase 2 (NOX2) is critical for ROS production by neutrophils and requires the formation of the NOX2 complex for activation, which comprises p47^phox^, p67^phox^, p40^phox^, and p22^phox^. *IL8* overexpression increased the mRNA levels of NOX2 complex members (Figure 3E). Phosphorylation of a stress kinase, p38, was also enhanced by IL8 overexpression (Figure 3F). Taken together, these findings suggest that IL8-driven neutrophil infiltration could enhance hepatic oxidative stress, presumably in association with neutrophil oxidative burst.

### 2.4. IL8 Overexpression Enhances Hepatic Inflammation in HFD-Fed Mice

**Figure 4:**
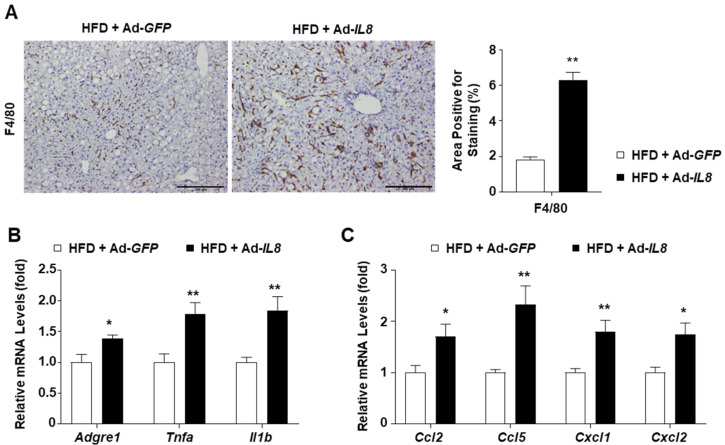
IL8 overexpression enhances hepatic inflammation in the livers of HFD-fed mice. (**A**) Paraffin-embedded liver sections were subjected to immunohistochemical analysis of F4/80 (*n* = 5/group). Representative images of F4/80 staining are presented (**left**). Scale bars indicate 200 μm. Quantification of the area positive for F4/80 staining is illustrated in graphs (**right**). (**B**,**C**) Mouse liver homogenates were subjected to RT-qPCR analysis of proinflammatory genes (panel **B**) and chemokine genes (panel **C**) (*n* = 5/group). Values represent the mean ± standard error of the mean (SEM). Statistical evaluation was performed using Student’s *t*-tests (* *p* < 0.05, ** *p* < 0.01). HFD, high-fat diet; IL8, interleukin 8; RT-qPCR, reverse transcription-quantitative polymerase chain reaction.

We further explored the inflammatory features in the livers of IL8-overexpressing mice to examine the effect of IL8 overexpression on the development of NASH-like phenotypes. In HFD-fed mice overexpressing IL8, F4/80 staining revealed increased formation of macrophage aggregates, known as hepatic crown-like structures, around lipid-laden hepatocytes, a hallmark of NASH-associated inflammation [31] (Figure 4A). Additionally, Ad-*IL8* infection increased the mRNA levels of inflammatory genes such as *Adgre1* (the gene encoding F4/80), *Tnfa*, and *Il1b* in the livers of HFD-fed mice (Figure 4B). Moreover, IL8 overexpression enhanced the mRNA expression of chemokine genes, such as *Ccl2*, *Ccl5*, *Cxcl1*, and *Cxcl2*, which induce monocyte and neutrophil chemotaxis (Figure 4C). Collectively, these findings demonstrate that IL8 overexpression could exacerbate hepatic inflammation, inducing the transition from fatty liver to NASH.

### 2.5. IL8 Overexpression Enhances Fibrosis in the Liver of HFD-Fed Mice

**Figure 5:**
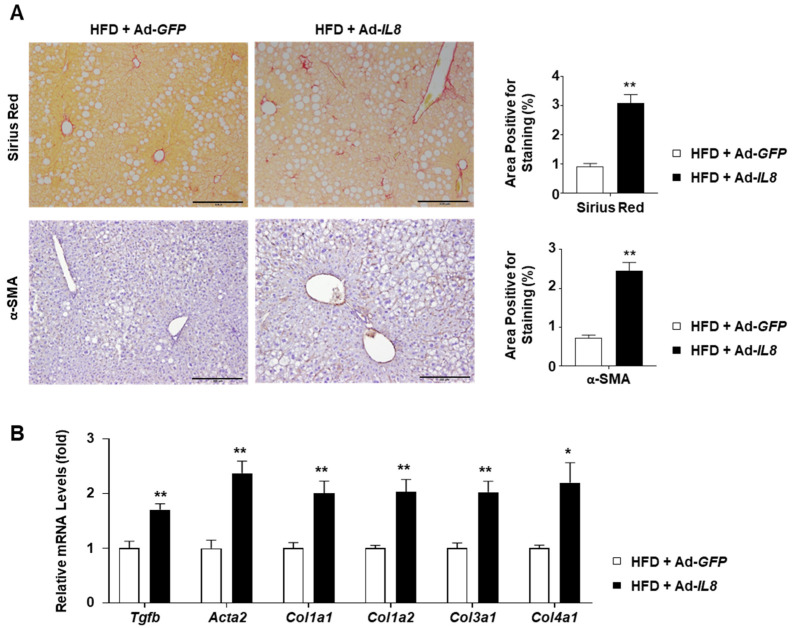
Fibrogenic effects of IL8 overexpression in the livers of HFD-fed mice. (**A**) Paraffin-embedded liver sections were subjected to Sirius Red staining and immunohistochemical analysis of α-SMA (*n* = 5/group). Representative images of each staining are presented (**left**). Scale bars indicate 200 μm. Quantification of the area positive for each staining is illustrated in graphs (**right**). (**B**) Mouse liver homogenates were subjected to RT-qPCR analysis of fibrogenic genes (*n* = 5/group). Values represent the mean ± standard error of the mean (SEM). Statistical evaluation was performed using Student’s *t*-tests (* *p* < 0.05, ** *p* < 0.01). α-SMA, alpha-smooth muscle actin; HFD, high-fat diet; IL8, interleukin 8; RT-qPCR, reverse transcription-quantitative polymerase chain reaction.

Having confirmed that IL8 overexpression promoted hepatic inflammation in HFD-fed mice, we subsequently explored whether these mice exhibited increased fibrosis. Sirius red staining of the mouse livers revealed that IL8 overexpression increased collagen deposition in the livers of HFD-fed mice (Figure 5A). Moreover, immunohistochemical analysis showed that Ad-*IL8*-infection increased levels of alpha-smooth muscle actin (α-SMA), a marker indicating the transdifferentiation of hepatic stellate cells into myofibroblasts. These histological findings were further substantiated by RT-qPCR analysis of key fibrogenic genes, including *Tgfb*, *Acta2* (encoding α-SMA), *Col1a1*, *Col1a2*, *Col3a1*, and *Col4a1* (Figure 5B). Collectively, our results suggest that IL8 overexpression in mice with fatty livers promotes fibrosis, a prominent characteristic of NASH.

### 2.6. IL8 Does Not Directly Stimulate Stress Kinase Activation or Fibrogenic Gene Induction In Vitro

**Figure 6:**
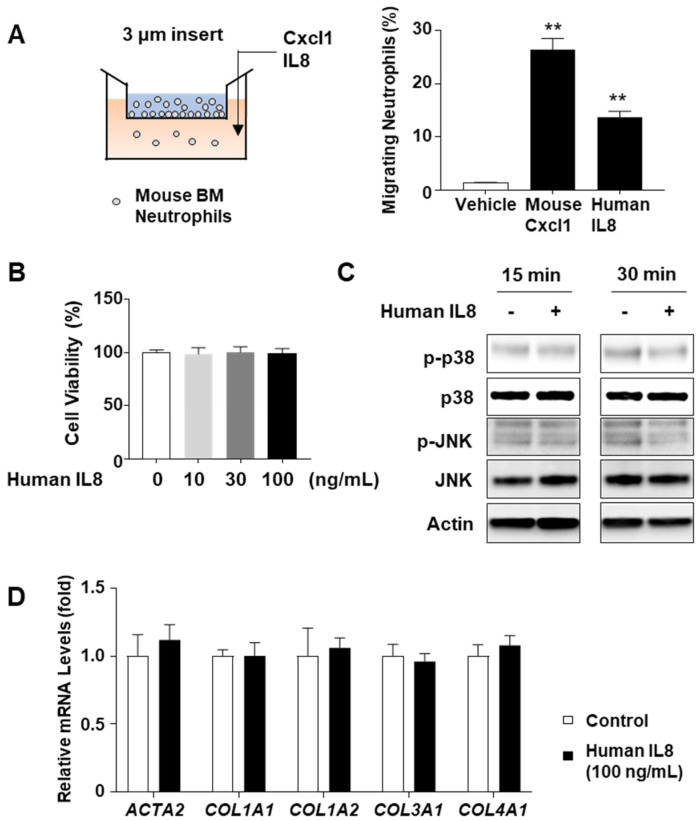
Effects of recombinant human IL8 protein on stress kinase activation and fibrogenic gene induction in vitro. (**A**) Schematic illustration of neutrophil chemotaxis assay. Mouse bone marrow (BM)-derived neutrophils were placed on the Transwell insert (pore size: 3 µm), and mouse Cxcl1 or human IL8 recombinant protein (100 ng/mL) was added in the bottom chamber. The number of neutrophils that migrated to the lower chamber over 6 h was counted and illustrated graphically (right). (**B**) AML12 mouse hepatocytes were treated with human IL8 recombinant protein (100 ng/mL) for 24 h and subjected to cell viability measurements using the CCK-8 reagent. (**C**) AML12 mouse hepatocytes were treated with human IL8 recombinant protein (100 ng/mL) for 15 min or 30 min and lysed in RIPA buffer for immunoblot analyses of stress kinases. (**D**) LX-2 human hepatic stellate cells were treated with human IL8 recombinant protein (100 ng/mL) for 24 h, and the total RNA was isolated for RT-qPCR analysis of fibrosis-related genes. Values represent the mean ± standard error of the mean (SEM). Statistical evaluation was performed using a one-way analysis of variance (ANOVA) with Tukey’s post hoc test for multiple comparisons or Student’s *t*-tests (** *p* < 0.01). HFD, high-fat diet; IL8, interleukin 8; RT-qPCR, reverse transcription-quantitative polymerase chain reaction.

Considering that mice lack the equivalent *IL8* gene found in humans, we investigated whether mouse neutrophils could respond to the human IL8 recombinant protein. The chemotaxis assay results showed that human IL8 stimulated neutrophil migration through Transwell chambers (pore size 3 μm), albeit to a lesser extent than the mouse Cxcl1 recombinant protein (Figure 6A). Based on this observation, we subsequently explored whether the NASH-associated characteristics observed in IL8-overexpressing mice could be directly attributed to IL8 itself, independent of infiltrating neutrophils. To verify this hypothesis, we treated AML12 hepatocytes with IL8 recombinant protein, subsequently evaluating the phosphorylation status of stress kinases, including p38 and JNK, which have been reported to be associated with hepatocyte death and activation in experimental NASH models [26]. However, IL8 treatment did not impact cell death (Figure 6B) or phosphorylation of p38 or JNK (Figure 6C). Similarly, treatment of LX-2 stellate cells with IL8 recombinant protein did not increase the mRNA levels of fibrogenic genes, such as *ACTA2*, *COL1A1*, *COL1A2*, *COL3A1*, and *COL4A1* (Figure 6D). In addition, treatment with mouse CXCL1 recombinant protein, the mouse homolog of IL8, failed to affect the phosphorylation of p38 or JNK in AML12 cells (Supplementary Figure S3). Accordingly, these findings suggest that the Ad-*IL8* infection-induced NASH progression might not be directly driven by IL8 but may involve other factors, such as neutrophil infiltration.

## 3. Discussion

Inflammation is a critical characteristic of NASH, which is not evident in a fatty liver. Thus, the factors responsible for initiating and propagating inflammation could be promising targets for combating the progression of fatty liver to NASH. Neutrophils are the first responders that initiate inflammation upon tissue damage, and an elevated neutrophil population is a hallmark of human NASH. Accordingly, neutrophils are considered crucial players in NASH progression.

The current study provides several lines of evidence demonstrating that IL8 overexpression in HFD-fed mice is a viable approach for investigating neutrophil-driven NASH progression in mice. Initially, we demonstrated that adenovirus-mediated overexpression of the human *IL8* gene increased serum and hepatic IL8 levels. Intravenously administered human adenovirus serotype 5 particles efficiently translocate to hepatocytes and Kupffer cells [32], which supports the notion that hydrodynamic delivery is useful for hepatic overexpression of genes of interest in mice. Additionally, we revealed that hepatic overexpression of human IL8 efficiently promoted neutrophil infiltration into the livers of mice despite species differences. To determine whether mouse neutrophils respond to human IL8, we performed in vitro experiments using a Transwell insert co-culture system. We observed that mouse bone marrow-derived neutrophils underwent chemotaxis in response to the human IL8 recombinant protein (Figure 6A). Our observations are consistent with those of previous reports, demonstrating that mice express a receptor homologous to human CXCR2 that induces signal transduction in response to human IL8 [33,34,35,36]. The considerable sequence homology between human and mouse CXCR2 proteins (71%, analyzed using NCBI BLAST) further supports the possibility that mouse neutrophils react with human IL8. Furthermore, IL8-induced hepatic neutrophil infiltration promoted liver injury, inflammation, and fibrosis in HFD-fed mice, indicating a transition from fatty liver to NASH.

CXCR2 activation induces events characteristic of neutrophil activation, such as the release of granule enzymes, ROS production, NETosis, intracellular Ca^2+^ influx, and chemotactic response [37,38], indicating that IL8 overexpression may not only stimulate hepatic neutrophil infiltration but also enhance the function of neutrophils. Using *Ncf1* knockout mice, we previously reported that Cxcl1-induced neutrophil-driven liver injury in HFD-fed mice was closely associated with a neutrophil oxidative burst mediated via the NADPH oxidase 2 complex [26]. We further revealed that IL-22 could mitigate Cxcl1 overexpression-induced liver injury in HFD-fed mice through a mechanism that required the induction of the antioxidant enzymes metallothionein-1 and metallothionein-2, thereby suggesting that attenuation of oxidative stress could ameliorate neutrophil-driven experimental NASH. Furthermore, the findings of the current study revealed that IL8-induced neutrophil infiltration could enhance oxidative stress and induce the expression of genes involved in the neutrophil oxidative burst.

Although we demonstrated that IL8 overexpression enhances oxidative stress in the mouse liver, the factors that mediate IL8- and neutrophil-induced NASH progression remain unclear. The ASK1–p38 axis is known to mediate ROS-dependent cell death [39], and we have previously implicated p38 in Cxcl1-induced liver injury and inflammation in hepatocyte-specific *Mapk14* (encoding p38 alpha protein) knockout mice [25]. However, *Mapk14* deletion failed to comprehensively reverse hepatic fibrosis. Thus, alternate mechanisms that may mediate neutrophil-driven NASH progression in the livers of IL8-overexpressing mice need to be considered.

One possible mechanism involves an interplay between neutrophils and stellate cells, as supported by several studies. For example, neutrophils can produce transforming growth factor β, the most critical cytokine that activates hepatic stellate cells [40]. Furthermore, neutrophils may stimulate stellate cells by producing ROS, whereas stellate cells prolong neutrophil survival, creating a positive feedback loop [41,42]. However, the role of the neutrophil–hepatic stellate cell interplay in NASH development remains poorly explored.

The function of neutrophil-derived factors in metabolic liver diseases has been extensively reported. Talukdar et al. have shown that hepatic insulin resistance and inflammation in HFD-fed mice were ameliorated following the deletion of the *Elane* gene, which encodes the neutrophil elastase protein, a neutrophil-secreted protease [43]. Moreover, Chen et al. reported that *Elane* knockout mice were protected against western diet-induced NASH, which was mediated via the regulation of ceramide metabolism [44]. Furthermore, *Mpo* knockout mice were found to be protected against diet-induced experimental NASH, indicating a role for MPO in NASH development [45]. Therefore, further exploration of the involvement of neutrophil-derived factors in IL8-induced NASH progression is necessary.

IL8 overexpression may directly contribute to NASH progression, independent of neutrophil infiltration. However, the current study provides evidence that negates the possibility of IL8 directly promoting liver injury or fibrogenesis. Our observation that treatment with IL8 recombinant protein failed to impact cell viability and the phosphorylation status of p38 and JNK in AML12 hepatocytes supports the notion that IL8 may not induce hepatocyte stress. Treatment with IL8 recombinant protein did not affect the mRNA levels of several fibrogenic genes. However, caution is needed when concluding that the IL8 protein may not directly promote NASH progression, as no comprehensive assessment of the effects of recombinant IL8 protein on all the factors involved in NASH pathogenesis was conducted in this study.

The present study corroborates the well-established concept that neutrophil infiltration is a hallmark of NASH, and neutrophils may contribute to the progression of fatty liver to NASH. In particular, we demonstrated the role of IL8, the most potent neutrophil-recruiting chemokine in humans, in the development of NASH, which has not been previously examined in experimental model organisms owing to interspecies differences in IL8 expression. It can be postulated that artificial overexpression of IL8 at high levels may perturb the physiological balance to an extent that deviates from the relevant pathway of NASH progression and may not replace existing NASH models. In this regard, the use of transgenic mice engineered to express the human *IL8* gene, including its regulatory elements, presents a promising avenue for understanding the impact of IL8 under conditions conducive to NASH development [46]. Nevertheless, the IL8 overexpression system can still effectively serve the overarching objective of the current study, i.e., to confirm that neutrophil infiltration, which is inefficiently induced in HFD-fed mice, is a crucial factor driving the progression of fatty liver to NASH. Furthermore, HFD feeding in conjunction with the IL8 overexpression system could be useful for studying the specific role of aberrant neutrophil infiltration in the pathophysiology of NASH.

Overall, the observations of the present study, along with those of our previous reports, support the conclusion that elevated expression of a single chemokine, such as CXCL1 or IL8, may facilitate NASH progression. This finding highlights the viability of targeting individual chemokines and/or their shared receptors (e.g., CXCR2) as a reasonable approach to mitigate NASH development. Future research should explore the therapeutic potential of the chemokine/receptor inhibition approach, as well as the identification of novel players involved in neutrophil biology during NASH progression.

## 4. Materials and Methods

### 4.1. Public RNA-seq Data Analysis

RNA-seq datasets of two independent human NAFLD patient cohorts were obtained from the Gene Expression Omnibus using the accession numbers GSE135251 and GSE167523. The metadata for each sample were downloaded using the R package ‘GEOquery’. The Wald test was used to assess changes in gene expression between the two groups (e.g., NASH vs. NAFL), which was implemented in the R package ‘DESeq2.’ Differentially expressed genes (DEGs) were selected based on cut-offs of false discovery rate (FDR)-adjusted *p*-value < 0.01 and |log_2_fold-change| > log_2_2.

To investigate the biological functions enriched in DEGs, over-representation analysis (ORA) was performed using gene ontology (GO) annotation. We employed a hypergeometric test to determine the statistical significance of the involvement of DEGs in each GO term, executed using the enrichGO function in the R package ‘clusterProfiler.’ The ORA generated multiple testing-corrected *p*-values (FDR-adjusted *p*-value or FDR) for each GO term.

To visualize the enriched GO terms and their relationships, we constructed a network (Supplementary Figure S1), with each node representing a GO term and the edges between nodes indicating the similarity between the GO terms. The size of each node corresponded to the number of genes involved in the respective GO terms, whereas the edge was based on a Jaccard coefficient ˃ 0.3. Consequently, similar GO terms with redundant functions were clustered in the network. The network was rendered using the EnrichmentMap app (v3.5) in Cytoscape software (v3.9.0).

### 4.2. Cell Culture

AML12 mouse hepatocytes were cultured in a 1:1 mixture of Dulbecco’s Modified Eagle Medium (DMEM) and Ham’s F12 medium containing 0.005 mg/mL insulin, 0.005 mg/mL transferrin, 5 ng/mL selenium, 40 ng/mL dexamethasone, 10% fetal bovine serum, and penicillin–streptomycin. LX-2 human hepatic stellate cells were cultured in DMEM supplemented with 10% fetal bovine serum and penicillin–streptomycin. Upon reaching 80% confluency, the cells were treated with human IL8 recombinant protein (R&D Systems, Minneapolis, MN, USA) for the indicated time to perform cell viability measurements, immunoblot analysis, and RT-qPCR. Following treatment, cell viability measurements, immunoblot analysis, and RT-qPCR were conducted, as described in the following sections.

### 4.3. Isolation of Mouse Bone Marrow-Derived Neutrophils

Mouse bone marrow was collected from the femur and tibia and passed through a 70-μm cell strainer in phosphate-buffered saline (PBS). The cell suspension was centrifuged at 300× *g* for 5 min, and the resulting leukocyte pellet was resuspended in RBC lysis buffer (Thermo Fisher Scientific, Waltham, MA, USA). After incubation on ice for 2 min, the cells were washed with PBS. Leukocytes were isolated using a Neutrophil Isolation Kit (Miltenyi Biotec, San Diego, CA, USA) according to the manufacturer’s instructions.

### 4.4. Neutrophil Chemotaxis Assay

Mouse bone marrow-derived neutrophils (2 × 10^6^ cells) were placed on an insert (3 μm pore size) in RPMI1640 media supplemented with 10% fetal bovine serum and penicillin–streptomycin. Recombinant human IL8 or mouse Cxcl1 protein was added to the medium in the bottom chamber. Migrating neutrophils were collected from the bottom chamber after 6 h and subjected to hemocytometer-assisted cell counting.

### 4.5. IL8 Overexpression in HFD-Fed Mice

C57BL/6J male mice (6–7 weeks of age) were fed an HFD (60 kcal% fat; D12492, Research Diets, New Brunswick, NJ, USA) or a chow diet (10 kcal% fat) for 3 months and administered 2 *×* 10^8^ PFU of Ad-*GFP* or Ad-*IL8* (Applied Biological Materials, Richmond, BC, Canada) via tail vein injection. The mice were fed an HFD or chow diet for 2 weeks after adenovirus infection until they were sacrificed for analysis. Animal experiments were performed in accordance with the NIH Guidelines for the Care and Use of Laboratory Animals, with protocols approved by the NIAAA Animal Care and Use Committee (approval number: LLD-BG-1).

### 4.6. Measurement of Serum ALT and AST Levels

Serum ALT and AST levels were determined in blood drawn from the mouse retro-orbital plexus using a Catalyst Dx Chemistry Analyzer (IDEXX Laboratories, Westbrook, ME, USA) according to the manufacturer’s instructions.

### 4.7. Enzyme-Linked Immunosorbent Assay (ELISA) Analysis

Serum levels of human IL8 were measured using a Human IL-8/CXCL8 Quantikine ELISA kit (R&D Systems, Minneapolis, MN, USA) according to the manufacturer’s instructions.

### 4.8. Histological and Immunohistochemical Analysis

Formalin-fixed liver samples were processed, and 4 µm thick paraffin sections were stained with Sirius Red dye (Sigma-Aldrich, St. Louis, MO, USA). For hematoxylin and eosin staining, paraffin-embedded sections were stained with hematoxylin and eosin (Thermo Fisher Scientific, Waltham, MA, USA). TUNEL staining was performed using an ApopTag^®^ Peroxidase In Situ Apoptosis Detection Kit (Millipore, Burlington, MA, USA). For immunohistochemistry, after heat-induced epitope retrieval, paraffin-embedded sections were incubated in 3% H_2_O_2_ and blocked in 3% normal serum buffer. The sections were incubated with primary antibodies overnight at 4 °C. The Vectastain Elite ABC staining kit and DAB peroxidase substrate kit (Vector Laboratories, Burlingame, CA, USA) were used to visualize the staining according to the manufacturer’s instructions. Immunohistochemical assessment was performed using primary antibodies against MPO (Biocare Medical, Concord, CA, USA), Ly6G (BioXCell, West Lebanon, NH, USA), MDA (Genox, Baltimore, MD, USA), 4-HNE (Genox), F4/80 (Cell Signaling Technology, Danvers, MA, USA), and α-SMA (Agilent DAKO, Santa Clara, CA, USA). Subsequently, we analyzed the positive cells and positive areas in 10 randomly selected high-power fields. The concentrations of antibodies used are described in Supplementary Table S2.

### 4.9. Immunoblot Analysis

The cells and mouse liver tissues were lysed in RIPA buffer containing a cocktail of protease and phosphatase inhibitors (GenDepot, Baker, TX, USA) according to the manufacturer’s instructions. Protein extracts were loaded onto 8% polyacrylamide gels and transferred to nitrocellulose membranes (Thermo Fisher Scientific, Waltham, MA, USA). Protein bands were visualized using Pierce ECL Western Blotting Substrate (Thermo Fisher Scientific, Waltham, MA, USA). Antibodies against p-p38 (Thr180/Tyr182), p38, p-JNK (Thr183/Tyr185), and JNK were purchased from Cell Signaling Technology. The concentrations of antibodies used are described in Supplementary Table S2.

### 4.10. Total RNA Isolation and RT-qPCR

Total RNA was purified from liver tissues or cell cultures using TRIzol reagent (Thermo Fisher Scientific, Waltham, MA, USA) according to the manufacturer’s instructions. One microgram of RNA was reverse transcribed into complementary DNA (cDNA) using a High-Capacity cDNA Reverse Transcription Kit (Thermo Fisher Scientific, Waltham, MA, USA). mRNA expression levels were measured via RT-qPCR using an ABI7500 RT-PCR system (Applied Biosystems, Foster City, CA, USA). GAPDH and APOB were used as internal controls. The 2^−∆∆Ct^ method was used to calculate mRNA levels. The primer sequences used for PCR are listed in Table 1.

### 4.11. Cell Counting Kit-8 (CCK-8) Assay

Cell viability was measured using a CCK-8 assay kit (Dojindo, Kumamoto, Japan) according to the manufacturer’s instructions. Briefly, AML12 mouse hepatocytes were treated with human IL8 recombinant protein (R&D Systems, Minneapolis, MN, USA) for 24 h. After the treatment period, the cells were further incubated with CCK-8 reagent for 2 h, and the absorbance was measured at 450 nm using a microplate reader.

### 4.12. Statistical Analysis

Data are expressed as the mean ± standard error of the mean (SEM) and were analyzed using GraphPad Prism software (v. 7.0a; GraphPad Software, La Jolla, CA, USA). Student’s *t*-tests were performed to compare the values obtained from the two groups. To compare more than three groups, a one-way analysis of variance was conducted, followed by post-hoc Tukey’s tests to determine specific group differences. A *p*-value of <0.05 was deemed statistically significant.

## 5. Conclusions

Based on the clinical observation that neutrophil infiltration is a hallmark of NASH that is not evident in fatty livers, the current study examined the contribution of IL8, a major neutrophil-recruiting chemokine, to the development of NASH in mice. Overexpression of IL8 in the livers of obese mice could accelerate the progression of fatty liver to NASH, along with a marked increase in liver injury, inflammation, and fibrosis. Further research is warranted to elucidate the detailed mechanisms through which IL8 overexpression contributes to NASH development.

## Figures and Tables

**Table 1:** Primer sequences for quantitative polymerase chain reactions.

Gene Name	Species	Forward (5′-3′)	Reverse (5′-3′)
*Acta2 (* *α-SMA)*	Mouse	TCCTGACGCTGAAGTATCCGATA	GGTGCCAGATCTTTTCCATGTC
*Adgre1 (F4/80)*	Mouse	CTTTGGCTATGGGCTTCCAGTC	GCAAGGAGGACAGAGTTTATCGTG
*Apob*	Mouse	CGTGGGCTCCAGCATTCTA	TCACCAGTCATTTCTGCCTTTG
*Ccl2*	Mouse	TCTGGACCCATTCCTTCTTGG	TCAGCCAGATGCAGTTAACGC
*Ccl5*	Mouse	GCTGCTTTGCCTACCTCTCC	TCGAGTGACAAACACGACTGC
*Col1a1*	Mouse	TAGGCCATTGTGTATGCAGC	ACATGTTCAGCTTTGTGGACC
*Col1a2*	Mouse	GGTGAGCCTGGTCAAACGG	ACTGTGTCCTTTCACGCCTTT
*Col3a1*	Mouse	TAGGACTGACCAAGGTGGCT	GGAACCTGGTTTCTTCTCACC
*Col4a1*	Mouse	CACATTTTCCACAGCCAGAG	GTCTGGCTTCTGCTGCTCTT
*Cxcl1*	Mouse	ACTGCACCCAAACCGAAGTC	TGGGGACACCTTTTAGCATCTT
*Cxcl2*	Mouse	CCAACCACCAGGCTACAGG	GCGTCACACTCAAGCTCTG
*Gapdh*	Mouse	AGCAGCCGCATCTTCTTGTGCAGTG	GGCCTTGACTGTGCCGTTGAATTT
*Il1b*	Mouse	TCGCTCAGGGTCACAAGAAA	CATCAGAGGCAAGGAGGAAAAC
*p22phox*	Mouse	ATGGAGCGATGTGGACAGAAG	TAGATCACACTGGCAATGGCC
*p40phox (Ncf4)*	Mouse	ATCGTCTGGAAGCTGCTCAA	CCCATCCATCTGCTTTTCTG
*p47phox (Ncf1)*	Mouse	TCCTCTTCAACAGCAGCGTA	CTATCTGGAGCCCCTTGACA
*p67phox (Ncf2)*	Mouse	TCTATCAGCTGGTTCCCACG	TGGCCTACTTCCAGAGAGGA
*Tgfb1*	Mouse	CAACCCAGGTCCTTCCTAAA	GGAGAGCCCTGGATACCAAC
*Tnfa*	Mouse	AGGCTGCCCCGACTACGT	GACTTTCTCCTGGTATGAGATAGCAAA
*ACTA2 (* *α-SMA)*	Human	GGAATGGGACAAAAAGACAGCTA	CGGGTACTTCAGGGTCAGGAT
*COL1A1*	Human	ACCTACAGCGTCACTGTCGATG	TTGTATTCAATCACTGTCTTGCCC
*COL1A2*	Human	TGGATACGCGGACTTTGTTG	CGGCTGGGCCCTTTCTTA
*COL3A1*	Human	TTGAAGGAGGATGTTCCCATCT	ACAGACACATATTTGGCATGGTT
*COL4A1*	Human	GGGATGCTGTTGAAAGGTGAA	GGTGGTCCGGTAAATCCTGG
*GAPDH*	Human	GCCCCAGCGTCAAAGGT	GGCATCCTGGGCTACACTGA
*IL8(CXCL8)*	Human	ACTGAGAGTGATTGAGAGTGGAC	AACCCTCTGCACCCAGTTTTC

## Data Availability

The data presented in this study are available on request from the corresponding author.

## Supplementary Materials

The following supporting information can be downloaded at: https://www.mdpi.com/article/10.3390/ijms242015489/s1.
