# Supplementary material for: Overexpression of Interleukin-8 Promotes the Progression of Fatty Liver to Nonalcoholic Steatohepatitis in Mice
Source: Int J Mol Sci. 2023 Oct 23;24(20):15489. doi: 10.3390/ijms242015489 (PMC10607753; doi:10.3390/ijms242015489)
Supplement: Supplementary file 1 [file ijms-24-15489-s001.zip › ijms-2653942-supplementary/Supplementary Figures (REVISED).pdf]

Supplementary Figure S1

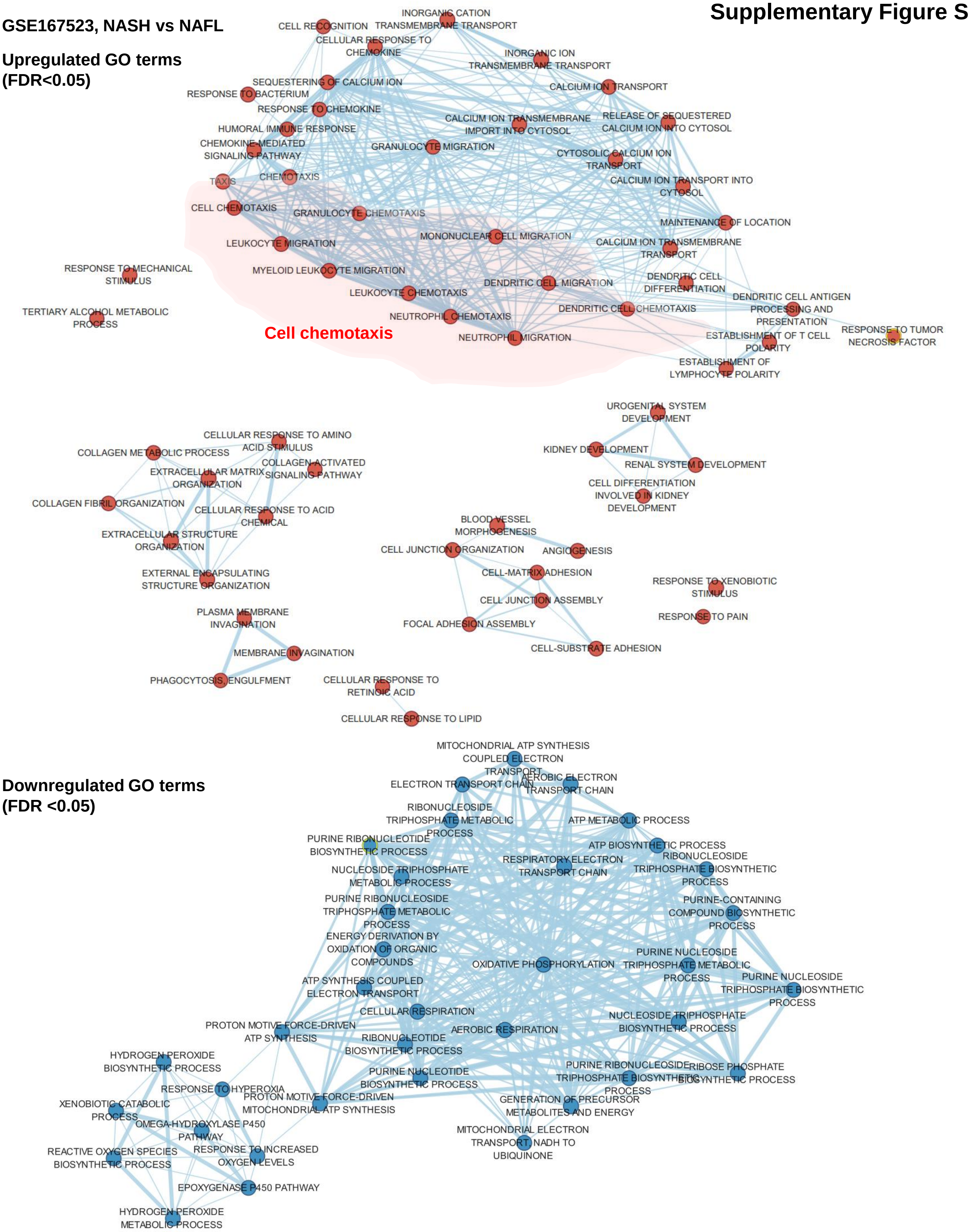

Supplementary Figure S1. Upregulated and downregulated gene ontology (GO) terms. Significant upregulation of genes involved in cell chemotaxis (highlighted in red) was observed in the livers of NASH patients compared with those of individuals with NAFL (obtained from a publicly available RNA sequencing database (GSE167523). FDR, false discovery rate.

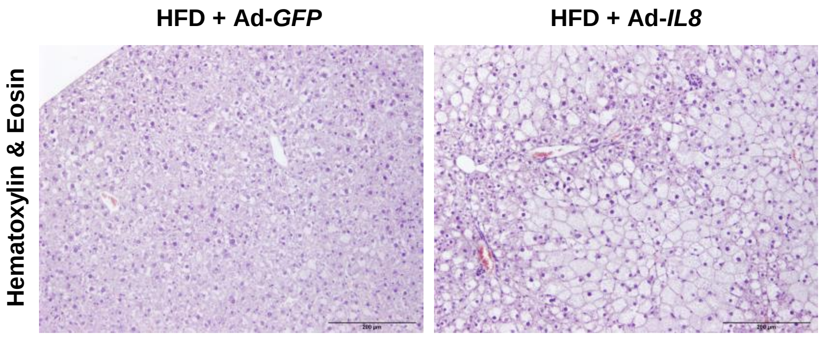

Supplementary Figure S2. Hematoxylin and eosin staining of the paraffin-embedded liver sections of mice fed an high fat diet (HFD) for 3 months and infected with Ad-*GFP* or Ad-*IL8* for 2 weeks. Scale bar, 200 μm

Supplementary Figure S3

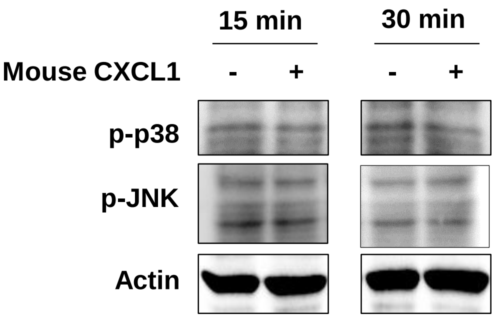

Supplementary Figure S3. Western blot analysis of phosphorylated p38 and JNK in AML12 hepatocytes treated with recombinant mouse CXCL1 (10 ng/mL) for 15 min or 30 min.
