# Supplementary material for: Overexpression of Interleukin-8 Promotes the Progression of Fatty Liver to Nonalcoholic Steatohepatitis in Mice
Source: Int J Mol Sci. 2023 Oct 23;24(20):15489. doi: 10.3390/ijms242015489 (PMC10607753; doi:10.3390/ijms242015489)
Supplement: Supplementary file 1 [file ijms-24-15489-s001.zip › ijms-2653942-supplementary/Supplementary Tables S1 and S2.pdf]

Supplementary Table S1. Formula for a high-fat diet (D12492, rodent diet with 60% kcal% fat)

**Ingredient**	**Gram%**	**Kcal%**
Protein	26.2	20
Carbohydrate	26.3	20
Fat	34.9	60

**Ingredient**	**Gram**	**Kcal**
Casein	200	800
L-Cystine	3	12
Corn Starch	0	0
Maltodextrin 10	125	500
Sucrose	68.8	275.2
Cellulose, BW200	50	0
Soybean Oil	25	225
Lard	245	2205
Mineral Mix, S10026	10	0
DiCalcium Phosphate	13	0
Calcium Carbonate	5.5	0
Potassium Citrate, 1 H2O	16.5	0
Vitamin Mix, V10001	10	40
Choline Bitartrate	2	0
FD&C Blue Dye #1	0.05	0
**Total**	**773.85**	**4057**

Supplementary Table S2. The concentration of antibodies used for immunohistochemistry and immunoblot.

Target Protein	Purpose	Dilution Ratio
MPO	Immunohistochemistry	Ready-to-use
Ly6g	Immunohistochemistry	1:300
MDA	Immunohistochemistry	1:400
4-HNE	Immunohistochemistry	1:400
F4/80	Immunohistochemistry	1:400
a-SMA	Immunohistochemistry	1:400
p-p38	Immunoblot	1:1000
P38	Immunoblot	1:1000
p-JNK	Immunoblot	1:1000
JNK	Immunoblot	1:1000
Actin	Immunoblot	1:1000
